# Low-dose spironolactone ameliorates adipose tissue inflammation and apoptosis in letrozole-induced PCOS rat model

**DOI:** 10.1186/s12902-022-01143-y

**Published:** 2022-09-07

**Authors:** Stephanie E. Areloegbe, Mmenyene U. Peter, Mosunmola B. Oyeleke, Kehinde S. Olaniyi

**Affiliations:** Cardio/Repro-Metabolic and Microbiome Research Unit, Department of Physiology, College of Medicine and Health Sciences, Afe Babalola University, P.M.B. 5454, Ado-Ekiti, 360101 Nigeria

**Keywords:** Adipose tissue, Inflammation, Oxidative stress, PCOS, Spironolactone

## Abstract

**Background of study:**

Globally, many reproductive aged women are affected by polycystic ovarian syndrome (PCOS), which is a common endocrine and metabolic disorder that is linked with adipose dysfunction and chronic low-grade inflammation. Spironolactone (SPL), a mineralocorticoid receptor blocker has been documented as a metabolic modulator. However, its immunomodulatory effect in PCOS is unknown. Therefore, the present study hypothesized that SPL would ameliorate adipose dysfunction and inflammation in experimental PCOS animals.

**Materials and methods:**

Female Wistar rats that were 8 weeks old were allocated into three groups. Group 1 received vehicle (distilled water; *p.o.*), group 2 received letrozole (1 mg/kg; *p.o.*) and group 3 received letrozole plus SPL (0.25 mg/kg, *p.o*.). The administration was performed once daily for 21 days.

**Results:**

The experimental PCOS animals showed insulin resistance, hyperinsulinemia and hyperandrogenism as well as oxidative stress and elevated inflammatory biomarkers (NF-kB/TNF-/IL-6) as well as a significant decrease in triglycerides, total cholesterol, free fatty acids, GSH and G6PD in the adipose tissue of PCOS animals. In addition, immunohistochemical assessment of adipose tissue showed significant expression of BAX and inflammasome, indicating apoptosis and inflammation compared to control animals. Nevertheless, administration of SPL attenuated these perturbations.

**Conclusion:**

Altogether, the present study suggests that low-dose spironolactone confers protection against adipose dysfunction in experimental PCOS animals by attenuating inflammation, oxidative stress and cellular apoptosis.

**Supplementary Information:**

The online version contains supplementary material available at 10.1186/s12902-022-01143-y.

## Introduction

Polycystic ovarian syndrome (PCOS) is the most common endocrine-metabolic disorder among women of reproductive age. The prevalence of PCOS ranges from 6–21% in reproductive aged women globally [1, 2]. It has been defined by the Rotterdam criteria with the presence of any two out of the following: oligomenorrhea, hyperandrogenism and polycystic ovaries [3, 4]. Polycystic ovarian syndrome has also been shown to be associated with some physiological changes such as insulin resistance, oxidative stress and psychological alterations like depression and anxiety [5, 6]. Evidence suggests that PCOS is a multifactorial disease, and individuals’ susceptibility is determined by genetic and environmental risk factors [7]. To date, the etiology of PCOS is not well established which makes the long-term effects such as cardiovascular diseases, type 2 diabetes, dyslipidemia and adipose tissue dysfunctions to be prominent [8–10].

Adipose tissue is regarded as an endocrine organ that plays a major role in the regulation of glucose/lipid metabolism and storage, with impact on energy expenditure, inflammation/immunity, cardiovascular function, and reproduction, among other functions [11]. Obesity is a major risk factor for insulin resistance and type 2 diabetes in PCOS and characterized with inflammation [12]. Chronic inflammation may involve persistent oxidative stress, thereby resulting in functional maladaptation, tissue remodeling and apoptosis [13, 14]. Adipose tissue remodeling is a constellation of visceral fat obesity, insulin resistance and atherogenic dyslipidemia, which all independently increase the risk of atherosclerotic diseases in PCOS individuals [15] and adipose dysfunction often contributes to the metabolic and reproductive phenotypes in PCOS women [16].

The pathophysiological mechanism of PCOS-associated adipose dysfunction is under elucidation. However, mineralocorticoid receptor (MR) activation has been shown to trigger abnormal responses in various tissues, including the adipose tissue [17, 18]. Similarly, obesity, type 2 diabetes mellitus, and other metabolic abnormalities are associated with activation of MR in the adipose tissue [19], while MR signaling is also involved in the normal physiological differentiation and maturation of adipocyte and enhanced activation of MRs contributes to insulin resistance, oxidative stress, pro-inflammatory adipokine and dysregulation of adipocyte autophagy as well as tissue apoptosis [19]. Spironolactone (SPL) is a MR blocker that regulates metabolic-related functions. Previous studies have demonstrated the protective effects of MR blockade by SPL against adipocytes cell injury [20] through reduction in insulin resistance [21]. Likewise, SPL is commonly used for the treatment of hirsutism and a drug of choice in the management of hyperandrogenism in PCOS individuals [22]. However, its use particularly at higher dose has been associated with intermenstrual bleeding due to reduction in production of estradiol and endometrial thickness [23, 24]. Interestingly, a number of studies, including recent studies from our laboratory animals have demonstrated the effectiveness and safety of low-dose SPL against endocrine disruption in PCOS models [25–28]. However, the effects of low-dose SPL on PCOS-associated adipose dysfunction is unknown. Therefore, the present study hypothesized that low-dose SPL would ameliorate adipose tissue dysfunction and inflammation in experimental PCOS animals.

## Materials and methods

### Design, grouping and treatment

The study was carried out and reported in accordance with the ARRIVE guidelines 2.0. Female Wistar rats that are 8 weeks old were procured from the animal house of the university, Afe Babalola University, Ado-Ekiti, Nigeria. The rats were kept under standard environmental conditions of temperature (22-26^0^C), relative humidity (50–60%), and 12-h dark/light cycle. These rats were given unlimited access to standard rat chow/tap water. The estrous cycles of the rats were determined through vaginal smear and all the animals used for this study were on the same estrous stage. The rats were acclimatized for 2 weeks and thereafter assigned into 3 groups of 6 rats per group: Group 1 is control, group 2 is letrozole (LE]T)-treated group and group 3 is LET + SPL-treated group. Group 1 received vehicle (distilled water, *p.o.*), group 2 received 1 mg/kg (*p.o.*) body weight of LET (Sigma-Aldrich, St Louis, MI, L6505), and group 3 received LET (1 mg/kg (*p.o.*) and SPL (0.25 mg/kg, *p.o*. Pfizer Limited, Kent, UK, C02DA01). The doses were selected as previously reported [27–30] and the administration was done uninterruptedly for 21 days.

### Plasma and adipose tissue sample collection

The rats were intraperitoneally anesthetized with 50 mg/kg of sodium pentobarbital as previously reported [28, 31] following an overnight fasting and determination of fasting blood glucose. Blood was collected by cardiac puncture into heparinized tube and centrifuged (704 g for 5 min) at room temperature. Plasma was stored frozen at -20 °C until the time of biochemical assays. After weighing the adipose tissue, 100 mg section of each tissue was carefully removed and homogenized with a glass homogenizer in phosphate buffer solution, centrifuged (8000 g for 10 min) at 4 °C and the supernatant was collected and stored frozen until the time of biochemical assays. Insulin resistance was determined using homeostatic model of assessment of insulin resistance (HOMA-IR) as previously described [28].

### Biochemical assays

#### Plasma insulin and testosterone

Insulin and testosterone concentrations were determined by Rat ELISA kits obtained from Calbiotech Inc. (Cordell Ct., El Cajon, CA 92,020, USA) while following the manufacturer's procedures.

#### Adipose lipid parameters

Standard colorimetric methods using assay kits obtained from Fortress Diagnostics Ltd. (Antrim, UK) with cat number BXC0271 for triglyceride (TG) and BXC0261 for total cholesterol (TC) were used to determine TG and TC from the supernatant of adipose tissue homogenates. Free fatty acid was determined using kit with cat number E-BC-K014 obtained from Elabscience Biotechnology Inc. (Wuhan, Hubei, P.R.C., China).

#### Adipose tissue malondialdehyde (MDA) and antioxidant markers

Malondialdehyde, reduced glutathione (GSH) and Glucose-6-phosphate dehydrogenase (G6PD) were determined from the adipose tissue by standard non-enzymatic and enzymatic spectrophotometric methods, using assay kits with cat number FR39 obtained from Oxford Biomedical Research Inc. (Oxford, USA) for MDA and GR2364/ PD380 for GSH and G6PD respectively obtained from Randox Laboratory Ltd. (Co. Antrim, UK).

#### Inflammatory biomarkers (NF-κB, TNF-α and IL-6)

The adipose levels of NF-κB, TNF- α and IL-6 were determined by quantitative standard sandwich ELISA technique using a monoclonal antibody specific for these parameters using rat kits with cat number E-EL-R0674, E-EL-R0019 and E-EL-R0015 respectively obtained from Elabscience Biotechnology Inc. (Wuhan, Hubei, P.R.C., China).

### Immunohistochemical assessment of adipose tissue using BAX and inflammasome (NLRP3) antibodies

Immunohistochemistry of adipose tissue was performed to detect the BAX and inflammasome antigens by the heat method of antigen retrieval as previously described (Olaniyi and Areloegbe, 2022) using BAX and NLRP3 antibodies with cat number E-AB-13814 and E-AB-65952) respectively obtained from Elabscience Biotechnology Inc. (Wuhan, Hubei, P.R.C., China). The BAX and NLRP3 are markers of apoptosis [32, 33] and inflammation [34] respectively. The slides were examined and captured using OPTO-Edu industrial camera light microscope and a computer (Nikon, Japan) and the representative of each group is shown in the results section. The expression and staining intensity of BAX and NLRP3 inflammasome were quantified in the adipose tissue by processing with an image-processing and analysis software Image-J (Version 1.52).

### Statistical analysis

All data were presented as means ± S.D. Statistical group analysis was performed with Graphpad Prim software version 5 with the use of One-way ANOVA to compare the mean values of variables among the groups, and post hoc analysis was performed with Bonferroni’s test. Statistically significant differences were accepted at *p* value less than 0.05.

## Result

### Effect of low-dose spironolactone on glucose/endocrine parameters in experimental PCOS animals

**Fig. 1:**
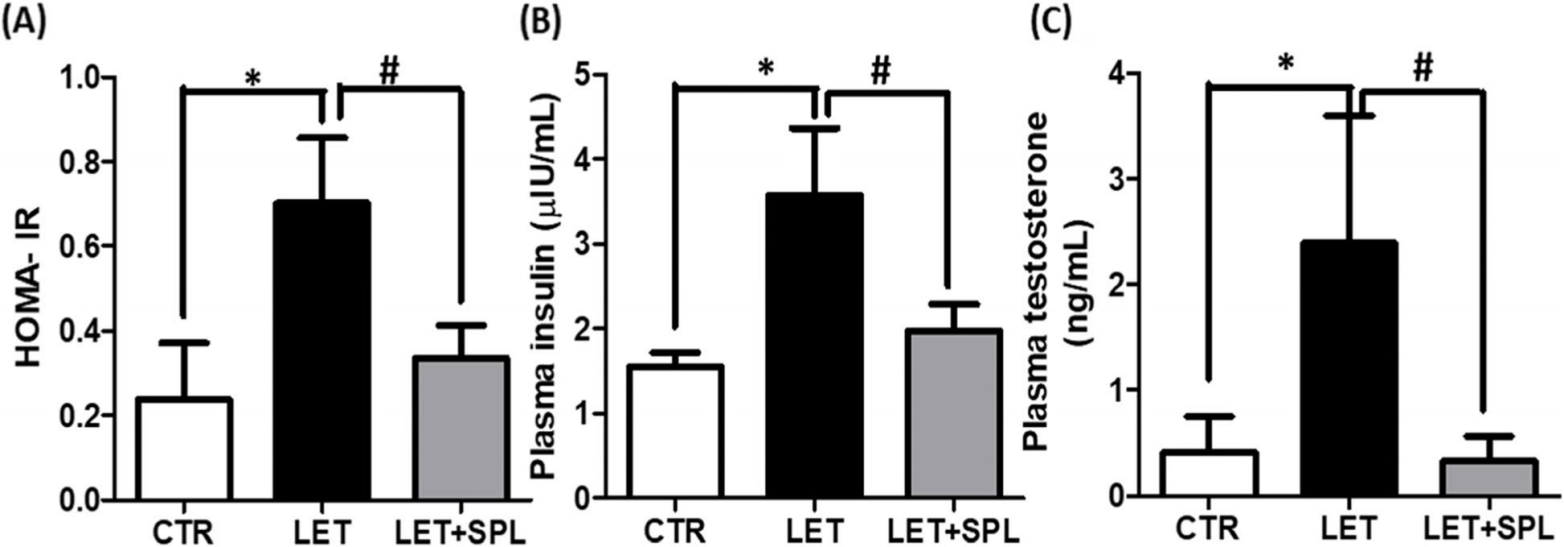
Effects of low-dose SPL on HOMA-IR (**a**), plasma insulin (**b**) and testosterone (**c**) in experimentally induced PCOS rat model. Data are expressed as S.D, *n* = 6. (*p < *0.05 vs. CTR, #p* < *0.05 vs. LET).* Control (CTR), Letrozole (LET), Spironolactone (SPL), Homeostatic Model of Assessment of Insulin Resistance (HOMA-IR)

There was a significant increase in HOMA-IR and plasma insulin and testosterone of PCOS animals compared with control and these were significantly decreased following the administration of SPL in PCOS animals compared with untreated experimental PCOS animals (Fig. 1).

### Effect of low-dose spironolactone on adipose lipid parameters in experimental PCOS animals

**Fig. 2:**
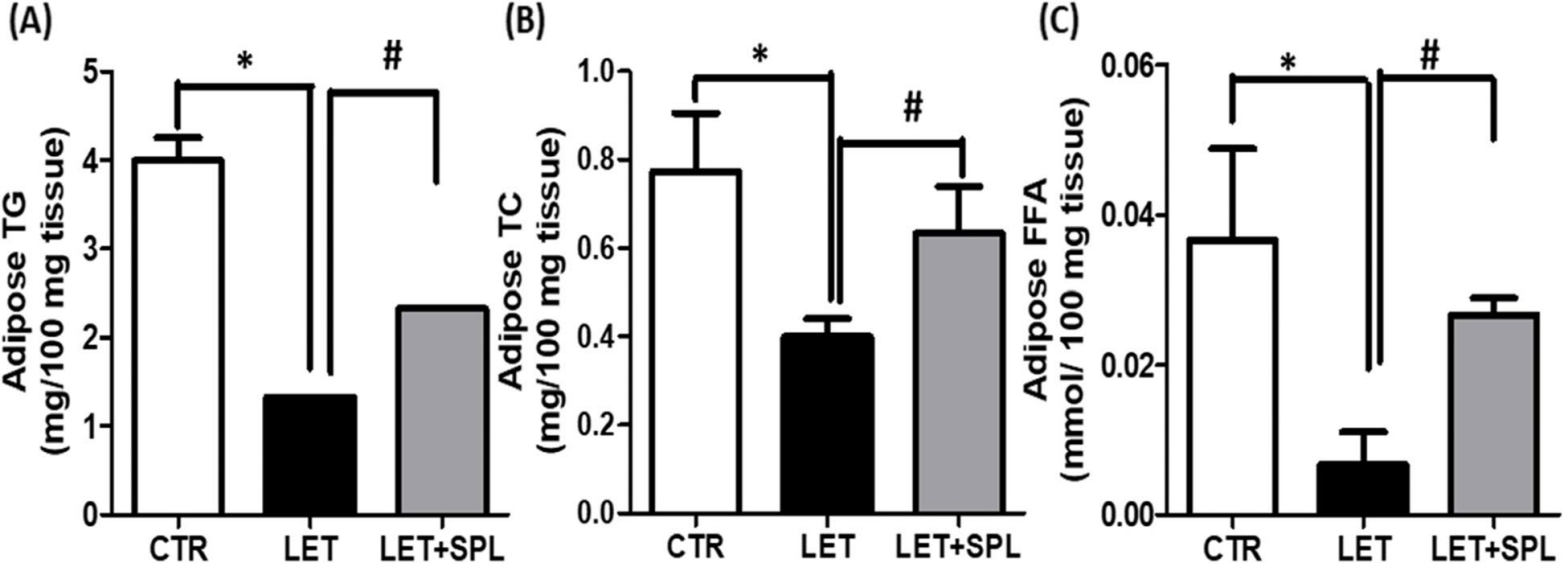
Effects of low-dose SPL on adipose TG (**a**) TC (**b**) and FFA (**c**) in experimentally induced PCOS rat model. Data are expressed as S.D, *n* = 6. (*p < *0.05 vs. CTR, #p* < *0.05 vs. LET).* Control (CTR), Letrozole (LET), Spironolactone (SPL), Triglycerides (TG), Total cholesterol (TC), Free fatty acids (FFA)

Lipid profile such as TG, TC and FFA, significantly decreased in the adipose tissue of experimental PCOS animals compared with control. Administration of SPL significantly increased the TG, FFA and TC of experimental PCOS animals compared with untreated experimental PCOS (Fig. 2, supplementary table 1, 2 and 3).

### Effect of low-dose spironolactone on oxidative stress markers and GSH/G6PD in the adipose tissue of experimental PCOS animals

**Fig. 3:**
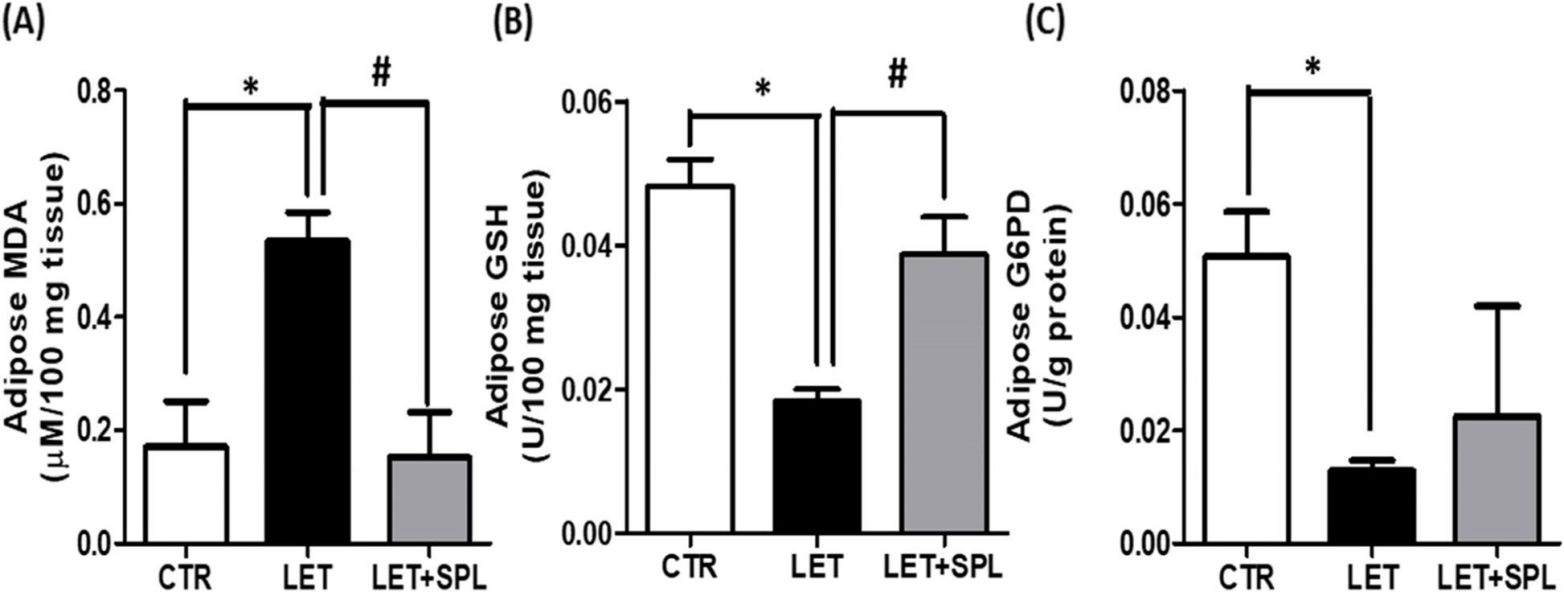
Effects of low-dose SPL on adipose MDA (**a**) GSH (**b**) and G6PD (**c**) in experimentally induced PCOS rat model. Data are expressed as S.D, *n* = 6. (*p < *0.05 vs. CTR, #p* < *0.05 vs. LET).* Control (CTR), Letrozole (LET), Spironolactone (SPL), Malondialdehyde (MDA), Reduced glutathione (GSH), Glucose-6 phosphate dehydrogenase (G6PD)

There was a significant increase in MDA while reduced glutathione and G6PD decreased significantly in the adipose tissue of experimental PCOS animals compared with the control. However, there was a significant reduction in the MDA levels and a significant increase in GSH but not G6PD levels following administration of SPL in experimental PCOS animals compared with untreated PCOS animals (Fig. 3, supplementary table 4, 5 and 6).

### Effect of low-dose spironolactone on inflammatory biomarkers in the adipose tissue of experimental PCOS animals

**Fig. 4:**
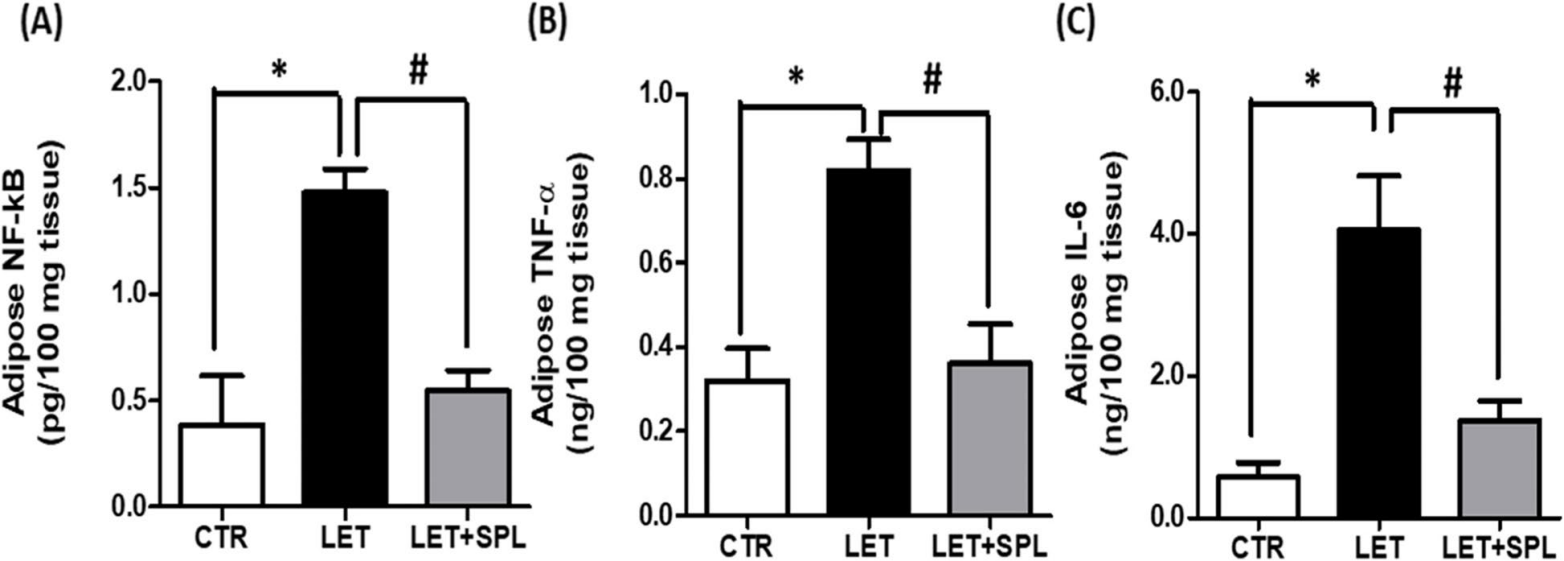
Effects of low-dose SPL on adipose NF-kB (**a**), TNF- (**b**) and IL-6 (**c**) in experimentally induced PCOS rat model. Data are expressed as S.D, *n* = 6. (*p < *0.05 vs. CTR, #p* < *0.05 vs. LET).* Control (CTR), Letrozole (LET), Spironolactone (SPL), Nuclear factor-kappa B (NF-kB), Tumor necrosis factor- (TNF-)

Inflammatory biomarkers such as NF-kB, TNF- and IL-6 increased significantly in the adipose tissue of experimental PCOS animals when compared with the control while administration of SPL significantly reduced these parameters in experimental PCOS compared with untreated PCOS animals (Fig. 4).

### Effect of low-dose spironolactone on immunohistochemical assessment of adipose tissue in experimental PCOS animals

**Fig. 5:**
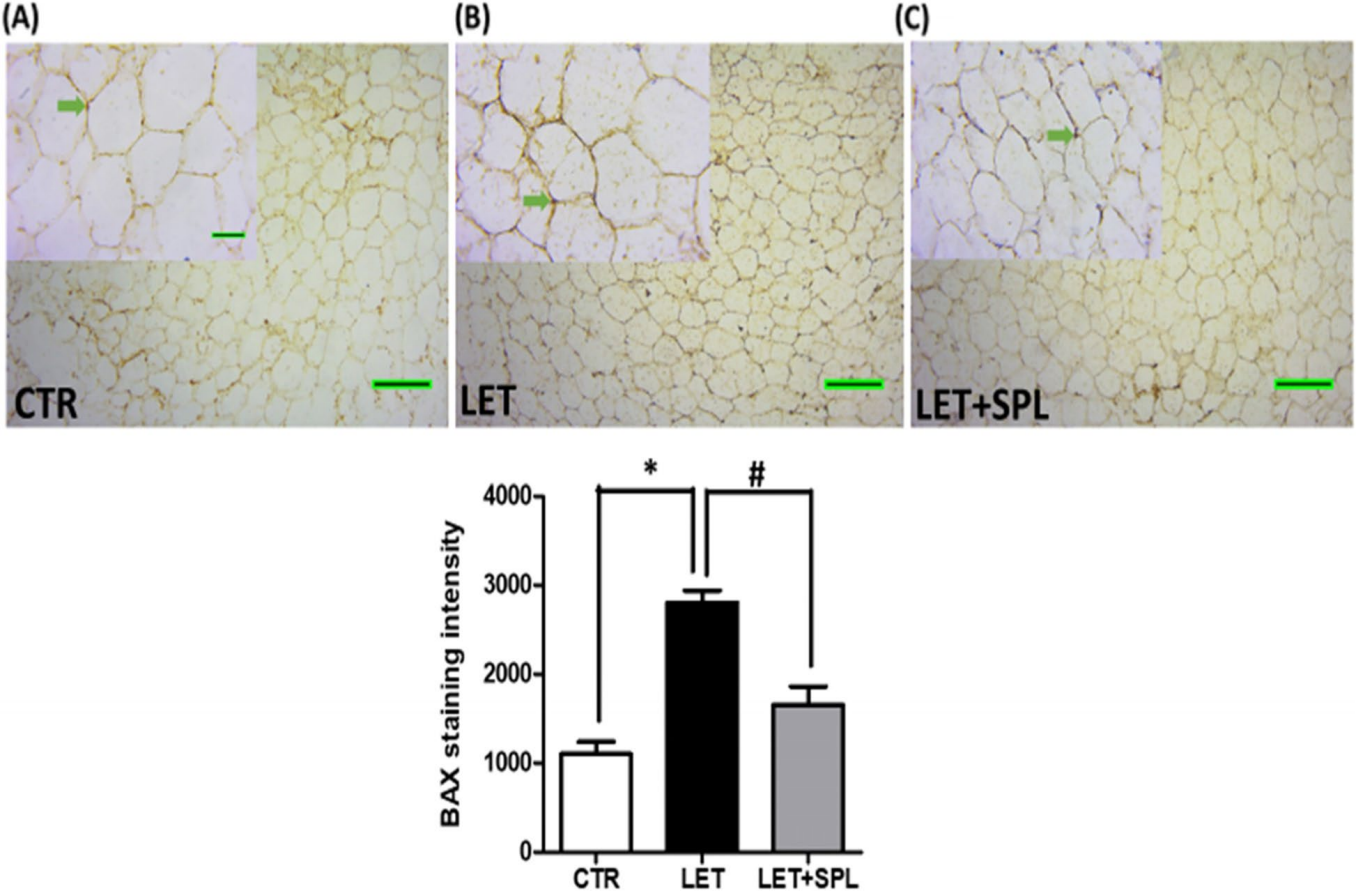
Effects of low-dose SPL on the immunohistochemistry of adipose tissue in experimentally induced PCOS rat model using BAX antibody. Scale bar: 51 μm. Small images (X200); Big images (X800). Data are expressed as S.D, *n* = 6. (*p < *0.05 vs. CTR, #p* < *0.05 vs. LET).* Control (CTR), Letrozole (LET), Spironolactone (SPL)

**Fig. 6:**
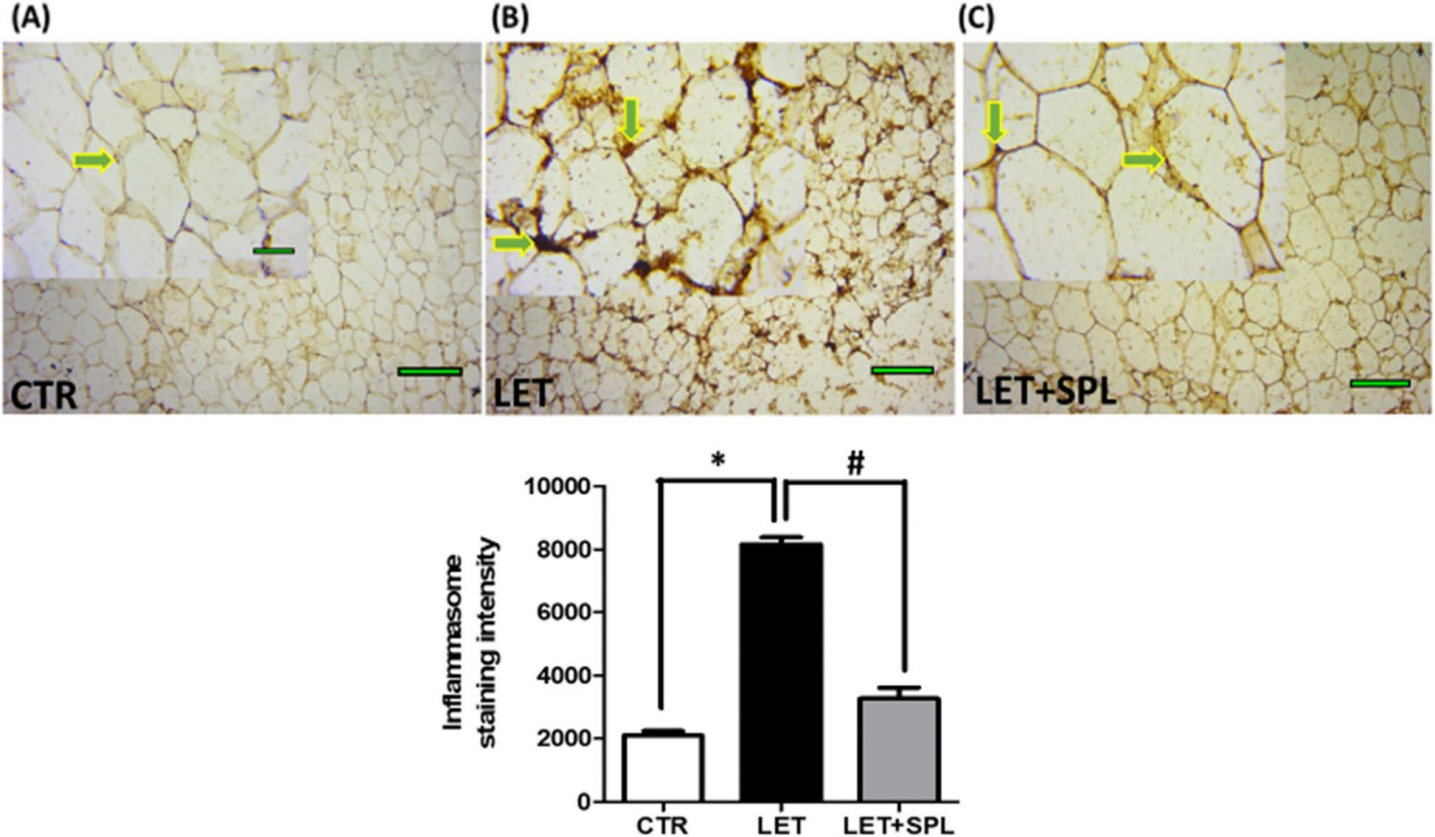
Effects of low-dose SPL on the immunohistochemistry of adipose tissue in experimentally induced PCOS rat model using NLRP3 inflammasome antibody. Scale bar: 51 μm; Small images (X200); Big images (X800). Data are expressed as S.D, *n* = 6. (*p < *0.05 vs. CTR, #p* < *0.05 vs. LET).* Control (CTR), Letrozole (LET), Spironolactone (SPL)

The adipose tissue of experimental PCOS animals showed significant expression of BAX and inflammasome indicating apoptosis and inflammation compared to control animals, which were attenuated in SPL-treated PCOS animals compared with untreated PCOS animals (Fig. 5 and 6).

## Discussion

The results of the present study demonstrate that low-dose SPL attenuates adipose dysfunction and/or inflammation/apoptosis in experimental PCOS rat model. The results in addition showed insulin resistance, hyperinsulinemia, hyperandrogenism and oxidative stress as well as a significant increase in inflammatory biomarkers (NF-kB/TNF- and IL-6), with a significant decrease in TG, TC, FFA, GSH and G6PD in the adipose tissue of PCOS animals. Besides, immunohistochemical evaluation of adipose tissue showed significant expression of BAX and inflammasome in experimental PCOS animals compared with control. Nevertheless, administration of SPL attenuated these alterations.

The results of the present study showed in PCOS animals, a significant increase in glucose-insulin index (HOMA-IR and plasma insulin) (Fig. 1a and b), compared with control group. Homeostatic model of assessment of insulin resistance is a surrogate marker of insulin resistance which is significantly high in animals with PCOS compared with control, validating altered metabolic phenotypes as an integral feature of PCOS. Hence, the present observation was consistent with previous studies that reported insulin resistance as a critical feature of PCOS [30, 35]. Insulin resistance in PCOS is likely to be caused by a post-receptor defect in insulin signaling, with increased serine phosphorylation and decreased protein kinase activity. This intrinsic defect in insulin receptor signaling in PCOS usually leads to hyperinsulinemia [36] as observed in the present results with elevated levels of plasma insulin (Fig. 1b) in PCOS animals compared with the control. Hyperinsulinemia often influences ovarian function as previously documented in various PCOS models [37]. In consonance with earlier results, hyperinsulinemia possibly increases androgen production with elevated level of circulating testosterone (Fig. 1c), validating a clinical manifestation of PCOS in this experimental model. This might contribute to premature luteinization of granulosa cells, thus causing granulosa cells disruption and impaired follicular development that characterize PCOS [38].

Furthermore, insulin resistance, a driver of metabolic complications often alters adipose function by increasing lipolysis, causing a reduction in lipid levels as revealed by decreased TG, TC, and FFA (Fig. 2) in PCOS animals compared with control. This facilitates influx of lipids into non-adipose tissue resulting in intramural lipotoxicity as previously reported [39]. Abnormalities in these lipid levels could indicate dyslipidemia which is a major complication of PCOS and its associated CVD [30]. Dyslipidemia in PCOS may be consistent with an insulin resistant state [40]. In the present study, the adipose tissues of PCOS animals showed elevated levels of inflammatory biomarkers (NF-kB/TNF- and IL-6) (Fig. 4) when compared with control animals. This possibly results in the recruitment of macrophages into the adipose tissue, aggravating inflammatory process and impairing adipose cell function and consequently leading to cellular apoptosis. Immunohistochemical evaluation showed a significant expression of inflammasome in the adipose tissue of PCOS animals compared with control (Fig. 6), suggesting, insulin resistance-induced adipose tissue inflammation. Hence, the present observations were consistent with previous findings that demonstrated macrophage infiltration and inflammation in the adipose tissue as well as deteriorated insulin resistance in models of metabolic-related diseases, including PCOS [41, 42]. Macrophages invasion has been shown to play a central role in adipose tissue remodeling, a condition that is characterized with adipocyte hypertrophy, increased angiogenesis, immune cell infiltration, and extracellular matrix overproduction with qualitative and quantitative alterations in adipocyte and eventually adipose dysfunction [13, 43, 44]. Therefore, the present results suggest the manifestation of adipose tissue dysfunction and/or inflammation in experimental PCOS animals.

Moreover, the present results also demonstrated that PCOS is associated with elevated levels of adipose lipid peroxidation (MDA) (Fig. 3a) which further led to a depletion in antioxidant capacity as revealed by decreased GSH- and G6PD-dependent antioxidant defense (Fig. 3b and c), promoting oxidative stress in PCOS animals compared with control. The present observation was consistent with earlier studies that reported a significant increase in oxidative stress markers in PCOS compared with the non-PCOS individuals and considered oxidative stress as a potential inducer of PCOS pathogenesis [45]. Oxidative stress is a major contributor to cellular apoptosis, this is confirmed in the present study by immunohistochemical analysis using BAX antibody and the result revealed a significant expression of BAX, indicating the manifestation of cellular apoptosis in the adipose tissue of PCOS animals compared with control (Fig. 5). Hence, the present findings overall suggest the development of insulin resistance-related inflammation and cellular apoptosis in the adipose tissue of PCOS animals.

Interestingly, administration of low-dose SPL attenuated insulin resistance (Fig. 1a), hyperinsulinemia (Fig. 1b) and hyperandrogenism (Fig. 1c) with corresponding decrease in adipose tissue inflammation (NF-κB, TNF-α and IL-6) (Fig. 4) and oxidative stress as well as improvement in antioxidant capacity (Fig. 3) and adipose lipid metabolism (Fig. 2) in PCOS animals compared with untreated PCOS group. In addition, low-dose SPL also attenuated inflammasome expression (Fig. 6) and cellular apoptosis (Fig. 5) in LET + SPL group compared with untreated LET group. The present observations are similar to previous studies that demonstrated improvement in glucose/lipid metabolism in experimental PCOS model [25, 27, 28] following treatment with low-dose SPL. Likewise, SPL has been reported to act as an insulin sensitizer in individuals with PCOS thereby alleviating lipolysis and in turn improves glucose uptake and energy homeostasis [46], which is validated with the present results. Previous studies have also revealed that SPL improves adipocyte dysregulation in obese model [44, 47, 48]. Nevertheless, the present results possibly add to the knowledge in the field by showing the ameliorative effect of low-dose spironolactone on adipose tissue inflammation and cellular apoptosis in PCOS rat model. However, the present study is not without a limitation in such that the molecular mechanisms underlying the beneficial effects of low-dose SPL on adipose tissue inflammation/ apoptosis in PCOS rat model was not investigated. Importantly, the results of this study possibly provide a justification for future investigation of molecular processes underpinning the protective effects of low-dose SPL on PCOS-associated adipose dysfunction and its related metabolic/endocrine complications.

## Conclusion

Altogether, the present results suggest that low-dose spironolactone confers protection against PCOS-associated adipose dysfunction by attenuating inflammation, oxidative stress and cellular apoptosis.

## Supplementary Information

**Additional file 1.**

## Data Availability

The data supporting the present study will be made available from the corresponding author on request.
