# Supplementary material for: Low-dose spironolactone ameliorates adipose tissue inflammation and apoptosis in letrozole-induced PCOS rat model
Source: BMC Endocr Disord. 2022 Sep 7;22:224. doi: 10.1186/s12902-022-01143-y (PMC9454226; doi:10.1186/s12902-022-01143-y)

**Table 1: Triglyceride**


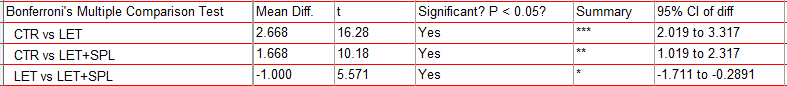


**Table 2: Total cholesterol**


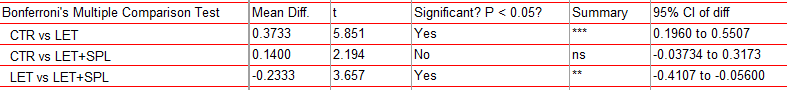


**Table 3: Free fatty acid**


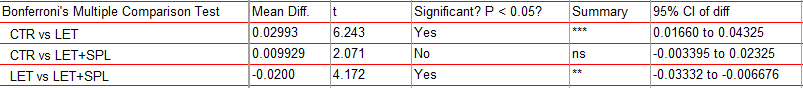


**Table 4: MDA**


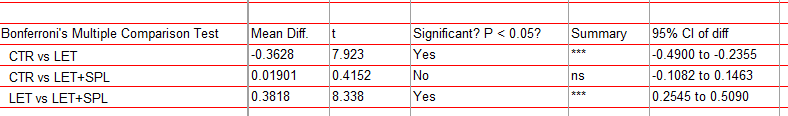


**Table 5: GSH**


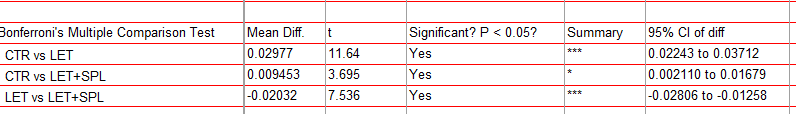


**Table 6: G6PD**


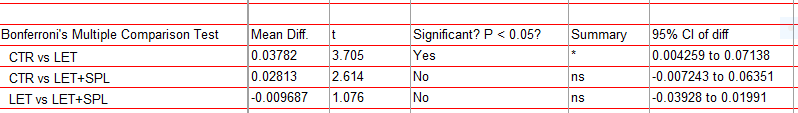

Supplement: Supplementary file 1 — Additional file 1. [file 12902_2022_1143_MOESM1_ESM.docx]
